# Editor’s Drawer

**Published:** 1892-04

**Authors:** 


### THE PAN-AMERICAN MEDICAL CONGRESS.

### EDITOR'S DRAWER.

The electro-gynecologists, having driven their batteries at a galloping pace through the female pelvis, are now turning their attention to a corresponding portion of the male anatomy. The prostate gland is the first object that arrests their attention, but we may expect soon to hear of the conquest of other organs in its vicinity.

---

The Medical Society of the State of New York, at its last meeting, appointed a committee of seven to coöperate in promoting the interests of the Pan-American Medical Congress in this State. The committee consists of Drs. A. Walter Suiter, of Herkimer; A. Vander Veer, of Albany; James D. Spencer, of Watertown; Seneca D. Powell, of New York; William Warren Potter, of Buffalo; D. B. St. John Roosa, of New York, and John O. Roe, of Rochester.

---

The *New York Medical Record* publishes a list of National and State medical societies, giving the names and addresses of the President and Secretary of each, together with the time and place of the next meeting. This furnishes a convenient reference for all interested in these societies, and entitles the *Record* to the thanks of the profession for its considerate thoughtfulness in this regard. We note an error in the announcement of the Medical Society of the State of New York in that the President this year is Dr. Lewis S. Pilcher, of Brooklyn. Dr. Suiter was the presiding officer during the last session.

---

The committee appointed at the last meeting of the American Medical Association to consider the best means for promoting the prosperity of the sections of the association, will hold an adjourned meeting in the Hotel Cadillac, Detroit, Mich., June 6th, at 3 P. M. Members of the committee are requested to notify the chairman of their intention to be present at this meeting. The committee would esteem it a favor if each member of the association would communicate in writing his or her views concerning the best measures for promoting the development of the sections. Such communications may be sent to the Chairman of the Committee, John S. Marshall, M. D., 9 Jackson street, Chicago.

---

The Committee on Public Health of the State Legislature, at a hearing held in Albany, February 24, 1892, listened to arguments pro and con relating to the baby Students' Bill No. 513. A very tame and weak opening was made on behalf of the students, which was followed by Dr. Edward Clark, of Buffalo, in opposition to the bill, who packed more solid logical argument into a fifteen minutes' speech than is common at such a hearing. He was followed by some other speakers, and the argument was finally closed by Dr. D. B. St. John Roosa, of New York, chairman of the legislative committee of the state medical society, who clinched the case with a few telling sentences that he is so capable of delivering. If there is anything in argument, or logic, or reason, it would seem as though the public health committee could not fail to be influenced by the earnest remarks of these gentlemen, who voiced the entire medical profession of the State of New York. The bill should slumber in the committee until the crack of doom.

---

NOMINATIONS were made by the Medical Society of the State of New York to fill vacancies on the State Board of Medical Examiners as follows: Dr. J. P. Creveling, of Auburn; Dr. Eugene Beach, of Gloversville; Dr. B. F. Sherman, of Ogdensburgh; Dr. A. Walter Suiter, of Herkimer. The term of service of Drs. Creveling and Beach will expire September 1, 1892, and from the four names submitted, as above stated, the Board of Regents will make appointments to fill these vacancies.

---

DR. R. FRENCH STONE, of Indianapolis, Ind., as editor and business manager for the publishers, Messrs. Carlon & Hollanbeck, announces the publication in the near future of a biography of eminent American physicians and surgeons. The work will be sold by subscription only, and it is proposed to limit the edition to between five and ten thousand copies, according to the number of subscriptions received. It is expected to complete the work before the close of the present year; hence it is desirable for all who wish the book to subscribe at an early date. The value of such a work cannot be questioned, as it enables one to readily refer to the status of a man whose personal acquaintance he may not have enjoyed. This and many other reasons conspire to make the book one which merits the support of the profession. The prices range from $8.00 to $10.00, according to the style of binding, and remittances may be made to Dr. Stone, 16 West Ohio street, Indianapolis, Ind.

---

THE average politician or capitalist has very little sympathy and less knowledge about the conduct of affairs in the public health service. Each of these classes generally thinks it a shrewd trick to keep the expenses of that department down to a minimum, which, judged by the standard of other departments, may be easily characterized as niggardly.

When Dr. Wende, the Health Commissioner, entered upon the duties of his office, he evidently did it with the intention of making the department what it should be in a great city like Buffalo. His recent visit to Indianapolis and Chicago, accompanied by Dr. Heath, the Food and Drug Inspector, will result in great good, we feel assured.

It is a strange revelation to find the city of Indianapolis, with a population less than one-half of that of Buffalo, appropriating several thousand dollars more annually to maintain something like a proper sanitary status. Chicago, only four times the size of Buffalo, maintains a health department at an expense of $600,000 annually, while the present officials of this city think $25,000 enough to allot this important department of the public service. Comment is unnecessary. The contrast is too striking to make it otherwise than painful to any physician who has the best interest of the city at heart.

---

